# 1‐D Collocated Dual‐Gradient Sensory Fibers for Comprehensive Sensing and Decoding of Complex Human Motion and Physiology

**DOI:** 10.1002/adma.74328

**Published:** 2026-07-27

**Authors:** Yunheum Lee, Sungha Jeon, Min Kim, Joonhee Won, Jinwook Yeo, Kyunghyun Jo, Soyeon Lee, Kieun Park, Yechan Yang, Chul Kim, Gun‐Hee Lee, Seunghwa Ryu, Sungho Jo, Seongjun Park

**Affiliations:** ^1^ Department of Bio and Brain Engineering Korea Advanced Institute of Science and Technology (KAIST) Daejeon Republic of Korea; ^2^ Medical Research Center Seoul National University Seoul Republic of Korea; ^3^ School of Computing Korea Advanced Institute of Science and Technology (KAIST) Daejeon Republic of KoreaKorea; ^4^ Department of Materials Science and Engineering Korea Advanced Institute of Science and Technology (KAIST) Daejeon Republic of Korea; ^5^ Department of Mechanical Engineering Korea Advanced Institute of Science and Technology (KAIST) Daejeon Republic of Korea; ^6^ Program of Brain and Cognitive Engineering Korea Advanced Institute of Science and Technology (KAIST) Daejeon Republic of Korea; ^7^ School of Electrical Engineering Korea Advanced Institute of Science and Technology (KAIST) Daejeon Republic of Korea; ^8^ Interdisciplinary Program in Bioengineering, College of Engineering Seoul National University Seoul Republic of Korea; ^9^ Departments of Biomedical Engineering Ulsan National Institute of Science and Technology (UNIST) Ulsan Republic of Korea; ^10^ School of Transdisciplinary Innovations Seoul National University Seoul Republic of Korea; ^11^ Department of Biomedical Science College of Medicine Seoul National University Seoul Republic of Korea; ^12^ Department of Transdisciplinary Medicine Seoul National University Seoul Republic of Korea; ^13^ Interdisciplinary Program in Neuroscience and Genetic Engineering College of Natural Sciences Seoul National University Seoul Republic of Korea; ^14^ AI Institute Seoul National University Seoul Republic of Korea

**Keywords:** artificial sensations, comprehensive sensory processing, dual‐gradient deep learning decoding, fiber sensor, stretchable electronics decoding

## Abstract

Wearable interfaces that capture both weak physiological fluctuations and large body motions remain difficult to realize in a single fiber because most strain‐sensing fibers are governed by one dominant electromechanical response mode. Consequently, mechanically distinct deformation regimes are ambiguously represented, limiting sensing range and information quality for downstream motion interpretation. Here, we develop a monolithic dual‐gradient fiber with complementary sensing regimes programmed through coupled materials and process design. By jointly tuning the percolation behavior and rheological drawability of carbon black/carbon nanotube‐filled styrene–ethylene–butylene–styrene (SEBS) composites, we identified two formulations suitable for coaxial co‐drawing within one continuous strand. The resulting fiber pairs a highly responsive layer for small deformation with a robust layer that stays informative at larger strain, producing synchronized but nonredundant signals. At low strain (0%–10%), the high‐sensitivity layer exhibits a gauge factor of 51.28, over four times the low‐sensitivity layer (12.42), while the two layers remain functional up to approximately 40% and 160% strain, respectively. This architecture preserves mechanically salient features across distinct and superimposed inputs, supporting measurements from pulse and respiration to joint motion. In a single‐fiber glove, the dual‐sensitivity design improves gesture‐classification accuracy by more than 10% relative to single‐sensitivity controls, enriching features for decoding.

## Graphical abstract

Coupled materials design enables a monolithic fiber that integrates complementary sensing regimes into a single wearable strand. By preserving informative signal features across subtle physiological deformation, large body motion, and mixed mechanical inputs, the dual‐gradient architecture generates synchronized, less redundant outputs that improve motion separation and downstream decoding in simplified wearable systems.

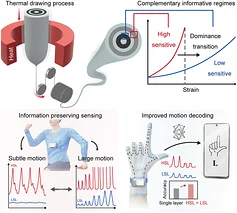

## 1 Introduction

Fiber‐shaped electronics represent an attractive materials platform for wearable sensing because their one‐dimensional geometry can be sewn, woven, or laminated onto curved and dynamically deforming body surfaces while preserving compliance and comfort. Consequently, strain‐sensing fibers have been widely explored for continuous monitoring of physiological signals, joint motion, and human–machine interactions [1, 2, 3, 4, 5, 6]. However, most existing fiber sensors are still governed by a single electromechanical transfer characteristic [7]. This creates a persistent trade‐off between sensitivity and operating range: devices optimized for minute deformation often provide strong signal amplification but saturate, drift, or fail under large strain [8, 9], whereas devices engineered for wide‐range motion lack the resolution needed to resolve weak physiological oscillations [10, 11]. For wearable systems that must interpret both subtle and large‐amplitude motions, the central limitation is therefore not only stretchability, but the inability of a single sensing mode to distinctly encode mechanically relevant features across disparate strain regimes [12, 13, 14].

A range of strategies has been pursued to address this limitation, including hierarchical conductive networks, structural microengineering, and multi‐sensor wearable layouts [15, 16, 17, 18, 19]. Although these approaches can improve task‐specific sensitivity or extend the usable strain window, most still rely on one dominant sensing channel at each local deformation site or on the combination of multiple discrete devices. In practice, this increases wiring, calibration, and mechanical complexity, while signals originating from different regimes may remain spatially separated or ambiguously mixed. The challenge is particularly acute in fiber platforms. Coating [20], electrospinning [21, 22], or related spinning processes [23] can produce multi‐component fibers, but heterogeneous layers formed through these approaches often suffer from weak interfacial adhesion, fatigue‐induced drift, limited radial compositional control, or poor scalability during textile processing [24, 25, 26]. More importantly, simply increasing the number of channels does not by itself solve the encoding problem, because multiple channels with similar transfer characteristics would still provide largely redundant outputs. What is required is not merely more channels, but collocated channels that preserve distinct and complementary information about the same mechanical event.

**FIGURE 1:**
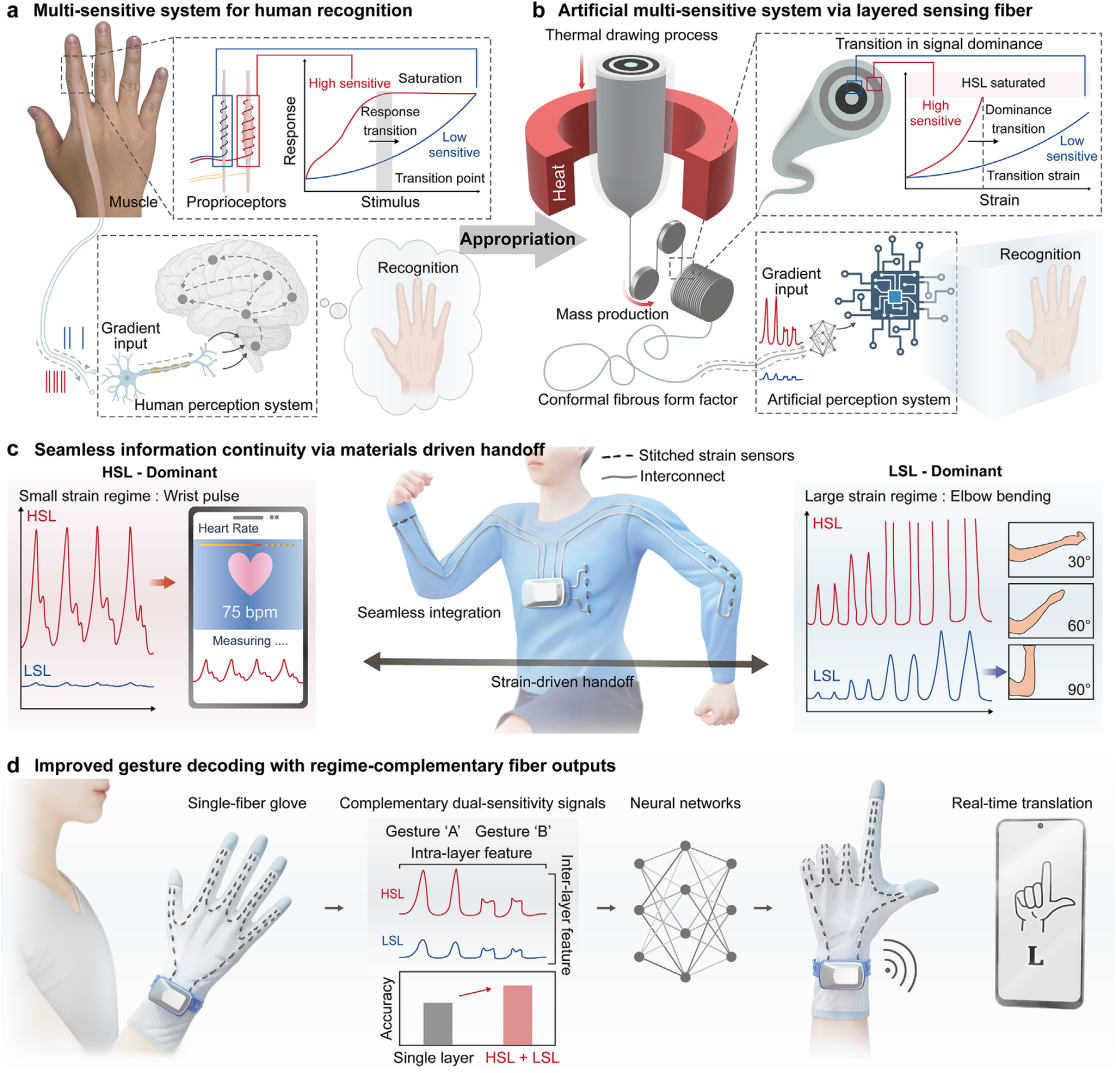
Concept and operating principle of the monolithic dual‐gradient fiber. a, Biological sensing concept with multiple sensitivity ranges. b, Thermally drawn collocated fiber with high‐ and low‐sensitivity layers showing strain‐dependent transition in signal dominance. c, Wearable sensing of low‐strain physiological signals and large‐strain body motion. d, Improved gesture decoding in a single‐fiber glove using dual‐sensitivity signals.

Biological proprioceptive systems suggest a different strategy [27, 28, 29, 30]. Rather than requiring a single receptor to remain equally informative across incompatible regimes, biological tissues distribute sensitivity across collocated elements whose relative contributions vary with stimulus magnitude (Figure 1a). In engineering terms, the key opportunity is a strain‐dependent redistribution of informative contribution between collocated sensing channels: one channel is more informative in the low‐strain regime, while another becomes more informative as deformation increases. Realizing this principle in an artificial fiber requires complementary sensing layers with deliberately differentiated transfer characteristics to be monolithically integrated within a single compliant body and to generate synchronized outputs that can be jointly interpreted. These requirements are difficult to satisfy using conventional multilayer fiber fabrication routes.

Thermal drawing provides an attractive route to address this challenge because it transforms a multi‐material preform into long, miniaturized fibers while preserving the designed cross‐sectional architecture, thereby enabling monolithic integration in a scalable format [31]. However, implementing multi‐regime sensing through thermal drawing is not simply a matter of stacking two strain‐sensitive composites with different gauge factors. In stretchable conductive composites, nanofiller concentration simultaneously governs electrical percolation, piezoresistive sensitivity, viscosity, viscoelasticity, and drawability [32, 33]. The realization of a monolithic multi‐sensitivity fiber therefore requires a coupled materials design strategy that jointly considers percolation‐controlled electromechanical response and rheological processability, rather than optimizing sensitivity alone. In other words, the relevant design space is not defined only by how strongly each layer responds to strain, but also by whether those differentiated layers can remain co‐drawable, structurally stable, and axially uniform within a single monolithic fiber.

Here, we report a composition‐programmed, thermally drawn dual‐gradient sensory fiber enabled by this rheology–percolation co‐design (Figure 1b). By tailoring the concentrations of carbon black (CB) and carbon nanotubes (CNT) in a styrene–ethylene–butylene–styrene (SEBS) matrix, we identify two co‐drawable piezoresistive layers with deliberately differentiated electromechanical behaviors and integrate them monolithically into a single continuous coaxial fiber. The resulting architecture combines a high‐sensitivity layer (HSL), positioned closer to the percolation threshold to resolve minute deformation, with a low‐sensitivity layer (LSL), containing a denser conductive network that remains informative under larger strain. Rather than merely broadening the measurable strain range, this radially programmed architecture generates complementary, temporally aligned dual‐channel outputs from the same deformation event. At low strain, the HSL provides the more informative signal; as deformation increases and the HSL loses useful dynamic range, the LSL becomes the dominant informative channel (Figure 1c). This strain‐regime‐dependent redistribution of informative contribution enables a single fiber to preserve mechanically salient features from subtle physiological motions to large joint excursions and to provide richer feature diversity and separability for artificial intelligence (AI)‐based motion decoding. We validate this concept through textile‐integrated monitoring of pulse and joint motion, as well as smart‐glove demonstrations of real‐time gesture decoding, including sign language translation, baseball pitch classification, and piano‐chord recognition (Figure 1d). Collectively, this work establishes thermally drawn dual‐gradient fibers as a scalable materials platform in which sensing performance, processability, and decoding‐relevant feature formation are co‐designed at the hardware level.

## 2 Results

### 2.1 Rheology–Percolation Co‐Design of Dual‐Gradient Sensing Fibers

To realize a monolithic dual‐gradient fiber capable of complementary mechanical encoding, the constituent sensing layers must satisfy two requirements simultaneously: they must exhibit deliberately differentiated electromechanical transfer characteristics while remaining compatible with a common thermal drawing process. We addressed this challenge using the thermal drawing process (TDP), in which a macroscale preform is thermally consolidated and elongated into a microscale fiber while preserving the prescribed cross‐sectional architecture (Figure S1 and Movie S1). This approach provides a scalable route for integrating multiple functional layers within a single fiber and avoids the delamination‐prone interfaces often associated with post‐assembled or sequentially fabricated structures (Figure S2). In the present system, TDP further enables discrete radial programming of filler concentration across coaxial layers, thereby providing a route to monolithically integrate collocated sensing channels with intentionally differentiated strain‐response regimes within one continuous fiber.

**FIGURE 2:**
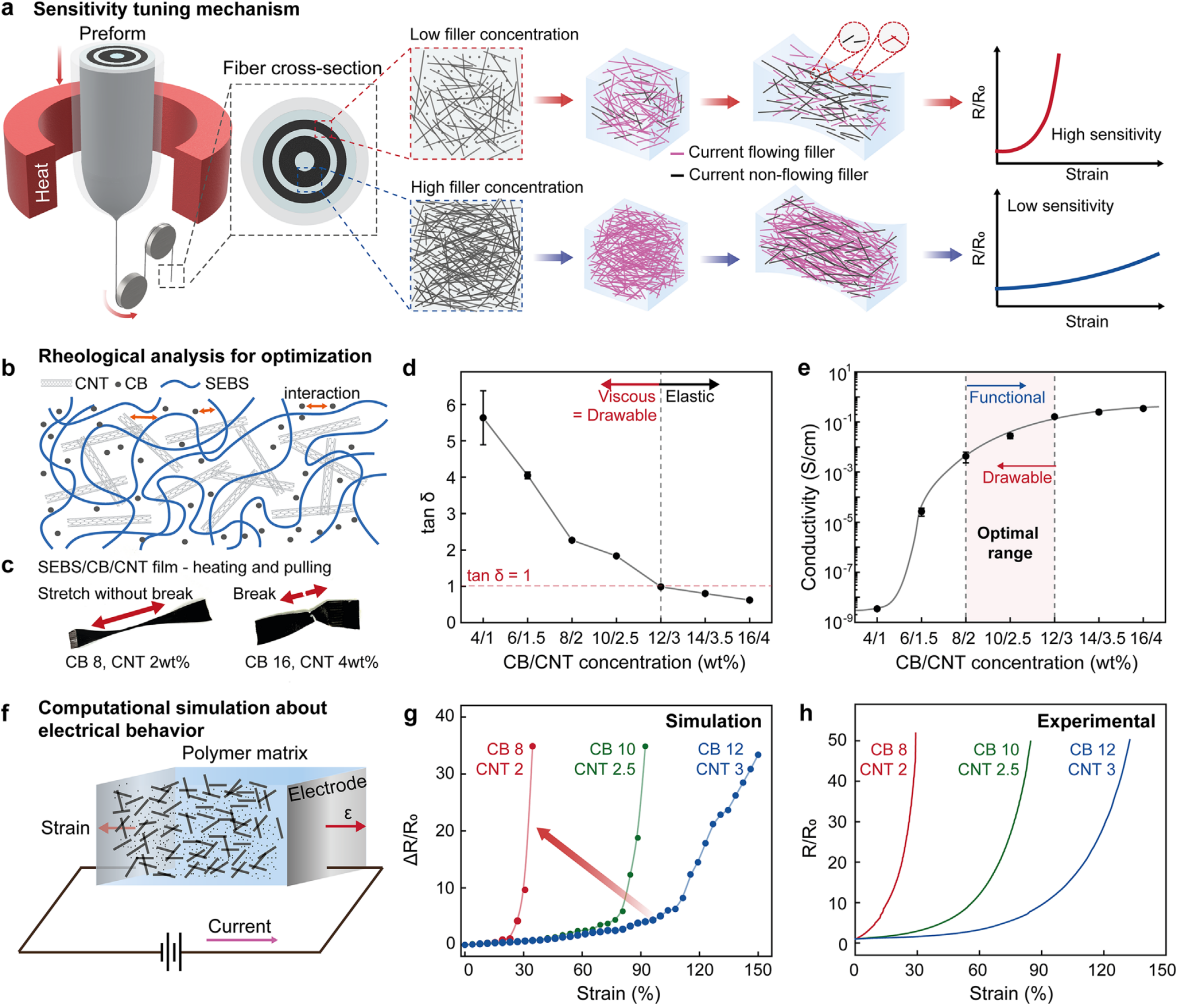
Rheology–percolation design and electromechanical tuning of dual‐gradient sensing layers. a, Schematic of filler‐concentration‐dependent sensitivity and representative resistance–strain responses. b, Schematic of CB/CNT filler interactions in SEBS. c, Stretching behavior of SEBS/CB/CNT composite films with different filler concentrations. d, Tan δ of SEBS/CB/CNT composites at 145°C as a function of filler concentration. Error bars indicate S.D. (*n* = 3). e, Electrical conductivity as a function of CB/CNT concentration, showing the co‐drawable sensing window. f, Conductive‐network model used for electromechanical simulation. g, Simulated resistance change as a function of strain for different filler concentrations. h, Experimental resistance change as a function of strain for different filler concentrations.

A key design parameter in this system is nanofiller concentration. In stretchable conductive composites, nanofiller concentration simultaneously governs electrical percolation, piezoresistive sensitivity, viscosity, viscoelasticity, and drawability. At relatively low filler contents, sparse conductive pathways disconnect under modest deformation, producing larger resistance changes and therefore higher strain sensitivity, but over a narrower useful operating window. By contrast, higher filler concentrations generate denser percolation networks that remain electrically connected to larger deformation, resulting in lower sensitivity but greater operational robustness (Figure 2a). This concentration dependence is further coupled to composite rheology, as polymer–nanofiller interactions, including van der Waals, electrostatic, and π–π interactions, alter the viscoelastic response of the melt (Figure 2b) [34, 35, 36]. As a result, filler loading must be selected not simply to maximize sensitivity, but to create co‐drawable layers with deliberately differentiated electromechanical transfer characteristics.

This coupling was evident in SEBS/CB/CNT composites, where drawability degraded as filler concentration increased (Figure 2c). Rheological measurements showed that the complex viscosity increased monotonically with filler loading, while tan δ decreased, indicating progressively reduced fluidity and greater elastic character at higher concentrations (Figures 2d and S3). Such rheological shifts are unfavorable for stable thermal drawing, particularly when the storage modulus approaches or exceeds the loss modulus, as observed at 12 wt% CB and 3 wt% CNT. In this regime, the reduced ability of the composite to undergo stable elongational flow complicates continuous fiber formation and undermines axial uniformity over long lengths. These results indicate that filler concentration cannot be tuned independently of processing constraints; rather, it defines a coupled rheology–drawability boundary that determines whether differentiated sensing layers can be monolithically realized in a common fiber body. Throughout this study, the CB:CNT mass ratio was fixed at 4:1. This ratio was selected based on the processability limits of the individual fillers, which were approximately 20 wt% for CB and 5 wt% for CNT in the present system (Figure S4). Maintaining a 4:1 ratio therefore kept both fillers within comparable fractions of their respective drawable limits while preserving thermal drawability. Holding the ratio constant also allowed total filler loading to serve as the primary design variable governing conductive‐network density and the resulting electromechanical response.

From an electrical perspective, the composite conductivity followed the expected percolation behavior, increasing sharply above the percolation threshold and approaching saturation at higher loadings [37, 38]. Experimentally, compositions above 8 wt% CB and 2 wt% CNT formed sufficiently conductive networks for sensing applications. By integrating these electrical and rheological constraints, we identified a practical co‐drawing window bounded by 8 wt% CB / 2 wt% CNT and 12 wt% CB / 3 wt% CNT (Figure 2e). Within this window, the two selected compositions provided both process compatibility and deliberately separated transfer characteristics, making them suitable as a high‐sensitivity layer (HSL) for minute deformation and a low‐sensitivity layer (LSL) for larger strain within a monolithic fiber architecture. Numerical simulations further supported this trend, confirming that reduced filler concentration increased the gauge factor by promoting earlier strain‐induced disruption of the conductive network (Figure 2f,g, S5, and S6) [39]. Collectively, these results establish a coupled materials‐design rule for thermally drawn dual‐gradient fibers, in which percolation‐controlled electromechanical differentiation and rheological drawability are co‐optimized to enable monolithic integration of collocated channels with complementary informative windows. This framework provides the materials basis for the strain‐dependent redistribution of informative contribution demonstrated in the following sections (Figure 2h).

### 2.2 Electromechanical Characterization of Dual‐Gradient Fibers

**FIGURE 3:**
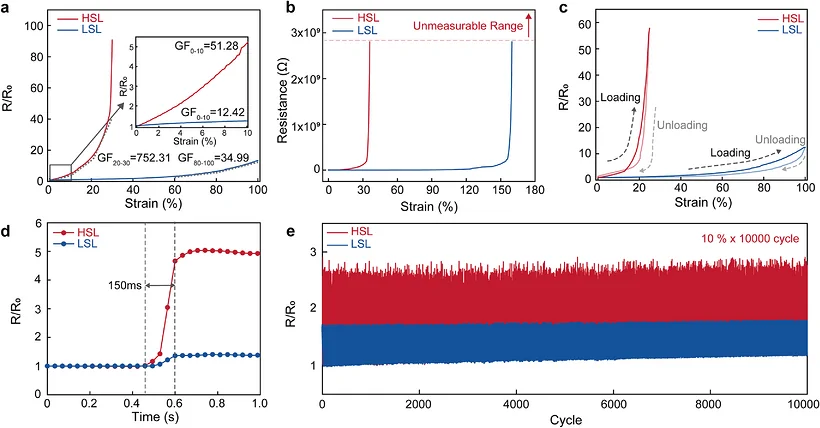
Electromechanical behavior and operational robustness of the dual‐gradient fiber. a, Resistance–strain responses of the HSL and LSL, showing higher sensitivity of the HSL and broader strain tolerance of the LSL. b, Working strain ranges of the HSL and LSL, defining the differentiated operating envelopes of the two layers. c, Loading–unloading hysteresis of the HSL and LSL within their respective strain ranges. d, Temporal response of each layer under 10% applied strain. e, Resistance stability over 10 000 stretch–release cycles (∼10% strain).

We next examined whether the coupled rheology–percolation design translated into differentiated yet coexisting electromechanical transfer characteristics within a single monolithic strand. To this end, we fabricated dual‐sensitivity strain‐sensing fibers comprising a high‐sensitivity layer (HSL; 8 wt% CB and 2 wt% CNT) and a low‐sensitivity layer (LSL; 12 wt% CB and 3 wt% CNT). Under low strain (0%–10%), the HSL exhibited a gauge factor of 51.28, more than four times that of the LSL (12.42), and this contrast became more pronounced with increasing strain, with the HSL gauge factor reaching 752.31 at 20%–30% strain before the layer approached the end of its useful range (∼40%). By contrast, the LSL maintained a stable and monotonic response over a broader usable deformation range, retaining a usable gauge factor of 34.99 at 80%–100% strain, which makes it more suitable for larger mechanical input (Figure 3a). These results confirm that the two collocated layers do not simply duplicate one another, but instead occupy distinct electromechanical operating windows and preserve different informative regimes within a single fiber.

The differentiated operating windows were further evident from the strain limits of the two layers. Under tensile deformation up to 200%, the HSL lost conductivity beyond ∼40% strain, whereas the LSL remained functional up to ∼160% strain (Figure 3b). This staggered operating envelope indicates that the two layers remain informative over different deformation regimes: the HSL preferentially resolves small strain, whereas the LSL preserves usable signal evolution under larger deformation. Upon release from overstrain, the resistance returned close to its initial value, indicating recovery of electrical continuity after temporary strain‐induced network disruption (Figure S7).

Reliable real‐time operation also requires low hysteresis and sufficiently rapid temporal response. Cyclic loading–unloading tests showed minimal hysteresis within the primary operational strain range, although hysteresis increased slightly beyond 100% strain after the elastic limit was exceeded (Figures 3c and S8a,b). The fibers also exhibited a response time of ∼150 ms, which is sufficient for dynamic physiological and kinematic monitoring (Figure 3d) [40, 41]. Additional frequency‐dependent cyclic measurements confirmed that the HSL remained well synchronized with applied deformation across the physiological and human‐motion frequency range, exhibiting only modest viscoelastic‐induced lag (Figure S9). Together, these results show that the differentiated transfer characteristics are maintained under repeated and time‐varying mechanical input.

For wearable integration, long‐term mechanical and environmental robustness are equally important. Thermal consolidation of the SEBS layers during TDP produced robust interlayer integrity, with no evidence of delamination over 10,000 tensile cycles (Figures 3e and S10). In addition, passivation layers shielded the fibers from environmental exposure, and immersion in water, detergent, ethanol, or acetone induced no significant electrical change (Figure S11). The temperature dependence of the dual‐gradient sensing response was also evaluated over the wearable‐relevant range of 20°C–40°C. Although both sensing layers exhibited modest baseline‐resistance variations with temperature, the relative sensitivity hierarchy was preserved, with the HSL remaining more sensitive in the low‐strain regime and the LSL maintaining a broader usable response at larger deformation (Figure S12). Collectively, these results demonstrate that the thermally drawn dual‐gradient fibers combine differentiated transfer characteristics, recoverable overstrain behavior, and operational robustness in a form factor suitable for wearable use. Rather than extending strain range through a single transfer characteristic, the architecture preserves distinct informative regimes through synchronized yet nonredundant sensing channels that remain operative over different deformation windows. This electromechanical characterization establishes the device‐level basis for the strain‐dependent redistribution of informative contribution examined under physiological, kinematic, and superimposed inputs in the following section.

### 2.3 Comprehensive Monitoring Across Distinct and Superimposed Deformation Regimes

**FIGURE 4:**
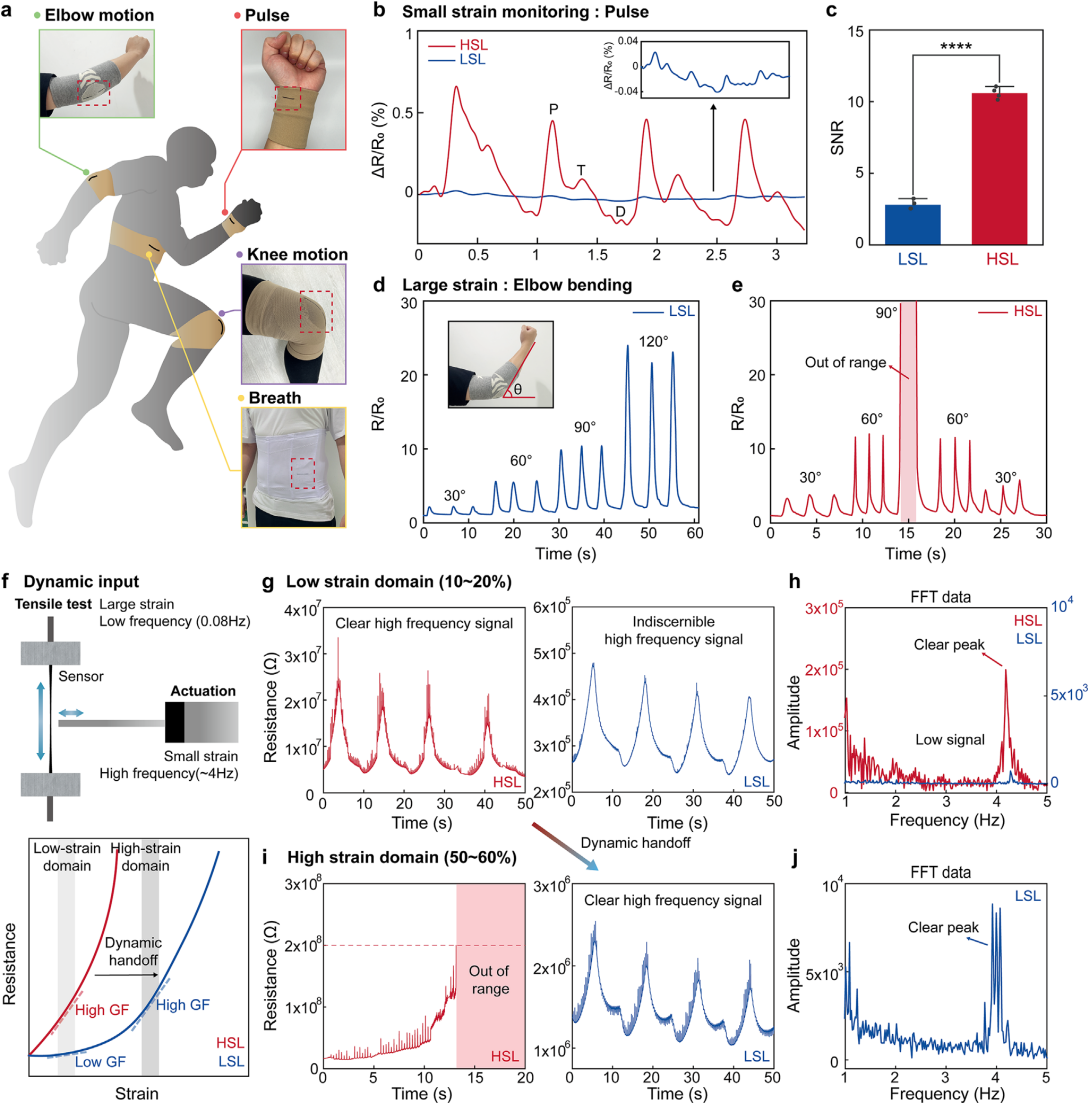
Multi‐regime wearable monitoring and dynamic handoff of the dual‐gradient fiber. a, Representative wearable measurement sites for physiological and kinematic monitoring using the dual‐gradient fiber, including pulse, elbow motion, knee motion, and breathing. b, Resistance change of the HSL and LSL during wrist pulse monitoring. The HSL resolves the percussion (P), tidal (T), and diastolic (D) peaks, whereas the LSL remains near baseline. **c,** Signal‐to‐noise ratio (SNR) of the HSL and LSL for pulse detection. Error bars indicate standard deviations (*n* = 3). Statistical significance was evaluated using a two‐sample *t*‐test (*p* < 0.0001). (d, e) Resistance changes during elbow bending measured by the d, LSL and e, HSL, showing extended operation of the LSL at large strain and earlier saturation of the HSL. f, Schematic of the dynamic input test and conceptual illustration of strain‐dependent nonlinear responses enabling dynamic handoff. (g, h) Time‐domain responses and FFT spectra in the low‐strain regime (10%–20%), where the high‐frequency component is resolved only by the HSL. (i, j) Time‐domain responses and FFT spectra in the high‐strain regime (50%–60%), where the HSL saturates and the LSL becomes the dominant informative channel.

To evaluate whether the differentiated transfer characteristics of the dual‐gradient fiber translate into practically useful wearable outputs, we integrated the fibers into elastic bands and textile garments for real‐time monitoring across physiological and kinematic regimes (Figure 4a). The one‐dimensional form factor and mechanical compliance of the fibers enabled conformal coupling to curved and dynamic body surfaces, while the toughness of the SEBS‐based matrix minimized damage during sewing and textile handling (Figure S13). This platform allowed us to examine not only whether the fiber spans a broad deformation range, but also whether the collocated layers preserve distinct informative features from the same body‐coupled input.

We first tested low‐strain physiological monitoring using a wristband configuration for radial artery pulse detection, which typically induces less than 5% strain. Under these conditions, the HSL reliably resolved the percussion (P), tidal (T), and diastolic (D) peaks, whereas the LSL remained near baseline (Figure 4b). This contrast was further supported by signal‐to‐noise ratio (SNR) analysis (Figure 4c). From the HSL waveform, we extracted cardiovascular parameters including beats per minute, systolic upstroke time, reflected wave transit time, H_T_/H_P_, and H_D_/H_P_ (Figure S14a,b) [42, 43], confirming that the high‐sensitivity layer is the more informative channel for subtle physiological deformation. To confirm long‐term reliability, wrist pulse was continuously monitored for 90 s (Figure S15a,b). The characteristic pulse waveform remained stable throughout the measurement. In addition, simultaneous measurements with a commercial PPG sensor showed good agreement in the extracted heart rate (Figure S15c,d), supporting the physiological relevance of the recorded pulse signal.

We next examined larger body motion. During elbow flexion, the LSL remained responsive over bending amplitudes beyond 120°, whereas the HSL provided useful output only up to ∼90° (Figure 4d,e). Additional calibration measurements confirmed that the sensor response could be used for quantitative joint‐angle estimation across the investigated range (Figure S16). A similar distinction was observed during respiration monitoring: the HSL more clearly resolved the small abdominal expansion associated with normal breathing, whereas the LSL remained informative during the larger expansion of deep breathing (Figure S17a,b) [44]. Simultaneous measurements using a commercial respiration‐belt reference further confirmed accurate extraction of breathing rate and respiratory waveform trends (Figure S18). Together, these results show that the two collocated layers preserve complementary informative regimes within a single monolithic fiber, enabling one strand to capture both subtle physiology and large‐amplitude motion without resorting to multiple discrete sensors.

We then asked whether the architecture could preserve useful information under superimposed input, in which a small, high‐frequency deformation is imposed on a large, low‐frequency background strain. To this end, a large‐amplitude 0.08 Hz actuation was combined with a small ∼4 Hz perturbation (Figure 4f). In the low‐strain regime (10%–20%), the HSL clearly resolved both the baseline deformation and the superimposed oscillatory component in the time domain, whereas the LSL showed little sensitivity to the high‐frequency feature (Figure 4g). Fourier analysis likewise revealed a distinct ∼4 Hz peak only in the HSL response (Figure 4h), indicating that the high‐sensitivity layer is the dominant informative channel in this regime.

By contrast, in the high‐strain regime (50%–60%), the HSL approached saturation and no longer resolved the superimposed oscillation, while the LSL became the more informative channel (Figure 4i). Importantly, this transition does not arise simply from the coexistence of two sensing layers, but from their deliberately differentiated transfer characteristics across strain regimes. As the background strain increased, the HSL lost useful dynamic range, whereas the LSL preserved informative signal evolution under larger deformation, allowing the previously unresolved high‐frequency component to emerge more clearly. The corresponding fast Fourier transform (FFT) of the LSL exhibited a distinct ∼4 Hz peak in this regime (Figure 4j), demonstrating a strain‐dependent shift in the dominant informative channel. Because the two channels encode the same input through different transfer characteristics, the small superimposed fluctuation remains resolvable through whichever layer is informative for the prevailing strain regime, with the HSL dominant at low strain and the LSL at high strain, rather than being masked by the large background. Thus, the dual‐gradient architecture preserves complementary signal structure across both distinct and superimposed inputs by redistributing informative contribution within one temporally aligned sensing strand.

Collectively, these results show that the monolithic dual‐gradient fiber extends beyond broad‐range sensing alone. Across pulse monitoring, large‐amplitude joint motion, and superimposed deformation, the two collocated layers preserve complementary informative regimes that cannot be simultaneously captured by a single transfer characteristic. By redistributing informative contribution according to the prevailing deformation regime, the architecture generates a richer and less redundant representation of the same mechanical event within a single wearable strand. This hardware‐level complementary encoding provides the basis for the improved motion separability and AI‐based decoding performance examined in the following section.

### 2.4 Single‐Fiber Glove for Gesture Decoding and Real‐Time Human–Machine Interaction

**FIGURE 5:**
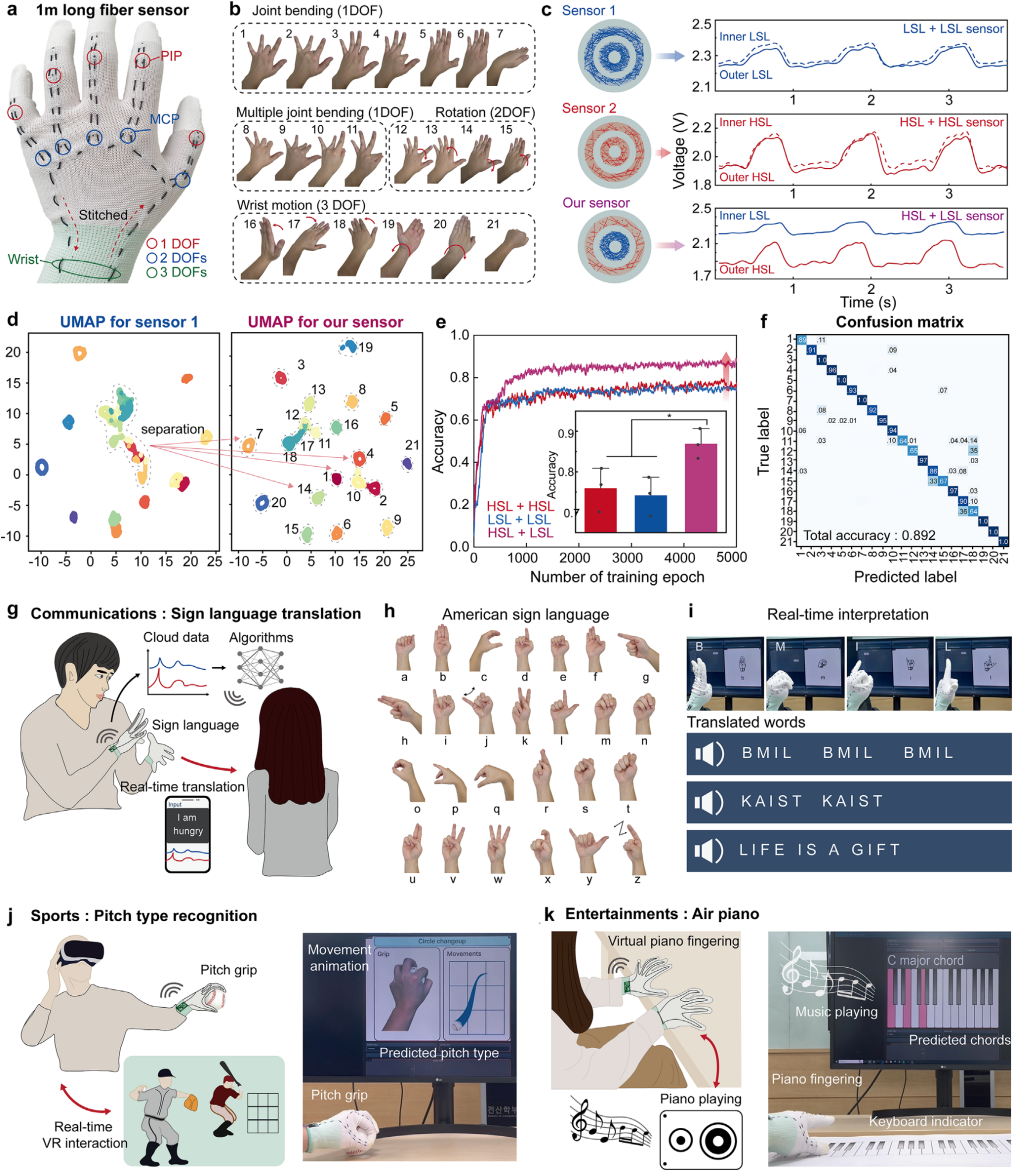
Complementary dual‐sensitivity signals improve gesture decoding in a single‐fiber glove and enable real‐time human–machine interaction. a, Smart glove integrating a single ∼1 m fiber that traverses the finger joints and wrist. b, 21 hand motions used for model training, including single‐joint bending, multi‐joint bending, rotation, and wrist motion. c, Comparison of sensor configurations and representative output waveforms for LSL+LSL, HSL+HSL, and HSL+LSL fibers under the same gesture input. d, UMAP visualization of the gesture dataset, showing improved class separation for the HSL+LSL sensor relative to a single‐sensitivity control. e, Classification accuracy as a function of training epoch for the three sensor configurations. The inset compares the final accuracies, showing improved performance for the HSL+LSL sensor. f, Confusion matrix for 21‐class gesture classification using the HSL+LSL single‐fiber glove. g, Schematic of sign language translation using the smart glove. h, American Sign Language alphabet used for model training and evaluation. i, Real‐time sign language interpretation and translated output. j, Sports application: pitch‐type recognition using the smart glove, including conceptual illustration and real‐time demonstration. k, Entertainment application: air‐piano interaction using the smart glove, including conceptual illustration and real‐time demonstration.

To determine whether the complementary outputs of the dual‐gradient fiber could improve motion interpretation in a simplified wearable format, we fabricated a smart glove by stitching a single 1 m fiber across the proximal interphalangeal (PIP) and metacarpophalangeal (MCP) joints of the fingers as well as the wrist, thereby spanning articulations with one, two, and three degrees of freedom (DOF) within the same sensing strand (Figure 5a) [45, 46]. The compliance and stretchability of the thermally drawn fiber enabled conformal integration across the complex topology of the hand without introducing bulky discrete sensing modules (Figure S19). Because conventional single‐sensitivity fiber sensors often struggle to distinguish similar signals arising from adjacent joints, this one‐strand glove provided a stringent test of whether a monolithic dual‐sensitivity architecture could improve motion separability despite minimal hardware complexity [47]. Using this platform, we collected time‐synchronized sensor data from 21 representative hand motions, including isolated joint bending, multi‐joint flexion, rotational gestures, and wrist movements (Figures 5b and S20) [48].

To isolate the effect of the dual‐sensitivity design, we compared three coaxial sensor configurations by varying the compositions of the inner and outer sensing layers: LSL+LSL, HSL+HSL, and HSL+LSL. For the same motion input, the LSL+LSL and HSL+HSL controls produced highly similar inner and outer waveforms, indicating substantial redundancy between the two channels (Figure 5c). By contrast, the HSL+LSL configuration generated differentiated yet synchronized outputs, reflecting the distinct transfer characteristics of the two layers under the same gesture. These differences were evident not only in signal amplitude, but also in the temporal evolution of the waveforms, indicating that the dual‐gradient architecture preserves complementary rather than duplicated information from a single mechanical event. To verify that the dual‐channel outputs retain mechanically meaningful information, we performed a single‐joint isolation analysis (Figure S21). Individual joints generated distinguishable response patterns and HSL‐to‐LSL ratios, reflecting the different strain regimes imposed on the fiber. These results indicate that the dual‐gradient architecture preserves joint‐dependent mechanical signatures that can contribute to downstream motion discrimination.

This complementary encoding was further reflected in the feature space of the gesture dataset. Uniform manifold approximation and projection (UMAP) revealed markedly improved separation among the 21 motion classes for the HSL+LSL sensor compared with the single‐sensitivity controls (Figure 5d). Whereas the LSL+LSL and HSL+HSL datasets showed substantial overlap between neighboring gesture classes, the dual‐gradient sensor yielded clearer clustering and larger inter‐class spacing, consistent with less redundant and more discriminative state information being retained in the dual‐channel output. These results indicate that integrating deliberately differentiated sensing regimes within one fiber can improve the separability of hand motions without increasing the number of sensing elements distributed over the glove.

To quantify this advantage, we trained a long short‐term memory (LSTM) network using the synchronized sensor outputs (Figure S22). The HSL+LSL configuration consistently converged to higher accuracy than either HSL+HSL or LSL+LSL and improved the final classification performance by more than 10% relative to the single‐sensitivity controls (Figure 5e). The corresponding confusion matrix showed an overall accuracy of 89.2% across the 21 gesture classes (Figure 5f). Similar trends were also observed across different model architectures (Figure S23), and comparable performance gains were reproduced for an independent participant (Figure S24), indicating that the decoding benefit arises from the complementary dual‐gradient encoding rather than from a particular learning model or user‐specific fitting.

To further demonstrate the translational utility of this platform, we adapted the same glove to real‐time human–machine interaction tasks spanning communication, sports, and entertainment. For communication, motion data corresponding to the 26 letters of the American Sign Language alphabet were used to train a task‐specific translation model [49], enabling real‐time decoding of letters, words, and short phrases (Figures 5g–i and S25; Movies S2 and S3). The system successfully distinguished visually similar hand shapes and translated examples such as “BMIL,” “KAIST,” and “LIFE IS A GIFT” in real time. In a sports setting, the glove was trained to classify five common baseball pitch grips together with a resting state (Figure 5j and S26), enabling real‐time discrimination of fine differences in hand posture relevant to pitch‐type recognition with an accuracy of 89.3% (Figure S27 and Movie S4). In an entertainment setting, the glove recognized hand configurations corresponding to piano chords and interfaced with a virtual piano system to provide visual and auditory feedback (Figure S28), achieving 88.6% accuracy and successfully reproducing chord progressions such as C–G–Am–F (Figure 5k and S29; Movie S5). Although these demonstrations are application‐specific, together they highlight the generality of a monolithic dual‐gradient fiber as a streamlined platform for motion capture, classification, and real‐time interaction.

Collectively, these results show that the value of the dual‐gradient architecture is not limited to broadening sensing range. By generating synchronized yet nonredundant outputs within a single compliant strand, the fiber improves motion separability and downstream decoding in a minimally instrumented wearable system. This capability is particularly relevant in the context of existing fiber‐based strain sensors, many of which achieve high gauge factors, broad working ranges, fast responses, or strong cyclic stability under specific testing conditions. A structured comparison with representative recent fiber‐based strain sensors is provided in Table S1. Rather than being defined by a single maximum sensing metric, the present platform is distinguished by combining textile‐compatible fiber geometry, monolithic preform‐to‐fiber co‐drawing, two electrically independent and collocated HSL/LSL channels, complementary low‐/high‐strain responses, cyclic and environmental robustness, and decoding‐relevant nonredundant signal generation within one continuous sensory fiber. These properties provide a practical route by which materials‐defined complementary encoding can reduce distributed sensing‐hardware complexity, including the number of sensing elements, wiring routes, and calibration points, while still supporting robust gesture recognition and real‐time human–machine interaction.

## 3 Conclusion

In summary, we establish a coupled materials‐design strategy for monolithic multi‐regime mechanical encoding by combining rheology–percolation co‐design with thermal drawing. By tailoring the carbon black/carbon nanotube composition of SEBS composites, we identified co‐drawable sensing layers with deliberately differentiated transfer characteristics and integrated them into a single continuous coaxial fiber. The resulting dual‐gradient architecture combines a high‐sensitivity layer for minute deformation with a low‐sensitivity layer that remains informative under larger strain, thereby enabling a strain‐dependent redistribution of informative contribution within one temporally aligned sensing strand. Quantitatively, the HSL exhibited a gauge factor of 51.28 at 0%–10% strain and remained functional up to ∼40% strain, whereas the LSL remained functional up to ∼160% strain and retained a gauge factor of 34.99 at 80%–100% strain. The fiber further exhibited a response time of ∼150 ms and stable resistance over 10,000 stretch–release cycles. Together, these characteristics support the broad‐range and complementary sensing capability of the dual‐sensitivity architecture. This materials‐defined complementary encoding preserved useful signal structure across physiological, kinematic, and superimposed inputs, supporting monitoring from arterial pulse and respiration to large joint motion. In a minimally instrumented glove, the synchronized yet nonredundant outputs improved motion separability and gesture‐decoding performance relative to single‐sensitivity controls, while enabling real‐time sign language translation, baseball pitch classification, and piano‐chord interaction. Rather than simply broadening sensing range, this work shows that differentiated transfer characteristics can be programmed into a monolithic fiber to shape the quality of information available for downstream interpretation. By co‐designing processability, electromechanical behavior, and feature formation within one scalable platform, thermally drawn dual‐gradient fibers provide a practical route toward next‐generation wearable interfaces for healthcare monitoring and real‐time human–machine interaction [50].

## 4 Experimental Methods

### 4.1 Materials

SEBS pellets (G1657 M) were purchased from KRATON. Polycarbonate (PC) films were acquired from SEOLIM AUTOMATION CO., LTD. CB (Carbon black Super P) and CNT (Multi‐walled) were purchased from Thermo Fisher Scientific and Sigma–Aldrich, respectively. CB demonstrated an individual particle diameter of ≲100 nm, while the CNT had a diameter of 50‐90 nm and an aspect ratio of 100. Tetrahydrofuran (THF) was purchased from Daejung Chemicals & Metals.

### 4.2 Preform Fabrication

The fabrication of the multi‐range sensitive fiber‐shaped strain sensor was achieved via the thermal drawing of a multi‐layered preform. The preform was composed of a pure SEBS core, insulation layers, CNT/CB/SEBS composite conductive layers, an additional insulation layer, and a PC sacrificial layer. The pure SEBS core was obtained by melting SEBS pellets and extruding them through a single screw extruder (Filastruder). The insulating films were fabricated by hot pressing the SEBS pellets. The conductive layer films were prepared using a solvent casting method. In this process, CB and CNT were dispersed in a 200 mL THF solution to create a suspension, utilizing a probe tip sonicator (VCX‐500, SONICS) for 30 min. Following this, 25 g of SEBS pellets were gradually added to the sonicated suspension and stirred at room temperature for 12 h, resulting in a conductive composite suspension. This suspension was then cast and dried under vacuum conditions at 60°C for 6 h, yielding conductive composite films. The films were subsequently pressed to a uniform thickness at 200°C under a pressure of 10 MPa for 10 min (Figure S30a). Finally, the conductive composite films, the insulation films, and a PC film were sequentially wrapped tightly around the pure SEBS core and consolidated in an oven at 220°C to complete the preform (Figure S30b).

### 4.3 Rheological Analysis

Rheological analysis was performed using a rheometer (Anton Paar, MCR 302). The polymer samples of each filler concentration were prepared in the form of a 1 mm thick, 8 mm diameter disc. The sample was loaded onto the Peltier plate and then heated to 160°C for 10 min, and placed in direct contact with the measurement plate without a gap. When the temperature of the Peltier plate was reduced to 152°C, the measurements were started and allowed to decrease at a rate of 1°C/min until 138°C, with a measurement taken every 1°C. 0.1% strain was applied to the sample at an angular frequency of 0.03 rad/s.

### 4.4 Thermal Drawing Process

TDP was performed in a custom‐built fiber drawing tower (Figure S1 and Movie S1). The preform is placed in the furnace and preheated to 140°C for 15 min. The furnace temperature was then increased to 150°C and waited for the elongation of the softened preform. When the fiber is hung on the capstan, the feed and capstan speeds are adjusted to 1 mm/min and 0.5∼1m/min to maintain constant tension by using a 3‐roller fiber tension meter (ZEF‐200, Hans‐Schmidt) to obtain the desired diameter fibers. Finally, the drawn fibers are chemically etched in dimethyl sulfoxide (DMSO) at 120 rpm in a shaker to produce a multi‐range sensitive fiber‐shaped strain sensor.

### 4.5 Measurement and Characterization

For measurement, separate tungsten wires (80 µm diameter, Goodfellow) were inserted into each sensing layer (HSL and LSL) and electrically connected with silver epoxy (CW2400, Chemtronics), and a thin layer of epoxy (5‐minute epoxy, DEVCON) was applied to fix each wire and sensor. Because the HSL and LSL are electrically isolated by the intervening SEBS insulation layer, each electrode pair contacts only its own layer, so the two layers are read out as independent channels acquired simultaneously rather than separated from a single combined signal by post‐processing (Figure S31). To evaluate the performance of the piezoresistive strain sensor, resistance was measured using a source meter unit (2450, Keithley). The voltage bias mode with the parameters set at a voltage level of 1 V and 1 mA of current limitation was used for measurement, and the sampling rate was set to 50 Hz. All electrical measurements were taken after the thermal drawing process, in the fiber form. A universal testing machine (LS1, Ametek) was used to accurately apply the desired strain and measure the mechanical properties of the strain sensor. A 10N load cell was used, and the stretching and releasing rate was fixed at 100% min^−1^ for each measurement. For the frequency‐dependent cyclic measurements, oscillatory tensile strain was instead applied by dynamic mechanical analysis (DMA; MCR 702e MultiDrive, Anton Paar) in frequency‐sweep mode from 0.5 to 9 Hz at a strain amplitude of 5%, while the electrical resistance of the HSL was recorded simultaneously.

Testing of the sensor response at different relative humidity was performed by simultaneously measuring the resistance of the sensor in a chamber where the relative humidity was increased using a commercial humidifier. The relative humidity in the chamber was measured using a wireless temperature and humidity data logger (TM‐305U, TENMARS). Chemical durability was performed by immersing the sensor in 100 mL of water, detergent, ethyl alcohol, and acetone for 10 min. The resistance of each layer was measured after overnight drying in a vacuum oven to eliminate interference from the ionic conductivity of the liquid. Temperature‐dependent electromechanical characterization was performed using a commercial temperature‐controllable refrigerator (0°C–65°C operating range). The sensing fibers were equilibrated at 20°C, 30°C, and 40°C prior to testing, and the actual chamber temperature was monitored using a handheld data‐logger thermometer (HH378 Data Logger, Omega). Resistance–strain measurements of both the HSL and LSL were then conducted under identical loading conditions at each temperature to evaluate the stability of the dual‐gradient sensing hierarchy under wearable‐relevant thermal conditions.

GF, Hysteresis, SNR, bpm, and MAE were calculated using the following equations.

$\mathrm{Gauge}\;\mathrm{Factor}\;\left(\mathrm{GF}\right)=\frac{\Delta R/R}{\varepsilon }$, where ∆R is the change in resistance, R is resistance, and ε is strain.

$\mathrm{Hysteresis}=\frac{A_{loading}-A_{unloading}}{A_{loading}}$, where A_loading_ is the area of the curve under loading state, and A_unloading_ is the area of the curve under unloading state.

$\mathrm{SNR}=10\;log_{10}\frac{R_{signal}}{R_{noise}}$, where R_signal_ is the peak resistance difference of the percussion wave peak and baseline, and R_noise_ is the root mean square of noise.

### 4.6 Physiological Measurements

For physiological‐signal validation, commercial reference devices were measured simultaneously with the fiber sensor. Pulse measurements were referenced using a photoplethysmography (PPG) sensor (PulseSensor Kit, World Famous Electronics llc.), while respiration measurements were referenced using a respiratory belt sensor (Go Direct Respiration Belt, GDX‐RB, Vernier Software & Technology). The physiological parameters extracted from the fiber sensor were compared with those obtained from the corresponding reference devices.

The instantaneous respiration rate was obtained from the time interval between successive inspiratory peaks of the band‐pass‐filtered (0.1–0.7 Hz) signal. For the *k*‐th peak detected at time *t_k_*, the instantaneous rate was calculated as *f_k_* = 60/(*t_k+1_−t_k_*) in breaths per minute. The instantaneous heart rate was obtained from the time interval between successive systolic (percussion, P) peaks of the band‐pass‐filtered (0.5–5 Hz) pulse signal. For the *k*‐th peak detected at time *t_k_*, the instantaneous rate was calculated as *f_k_* = 60/(*t_k+1_−t_k_*) in beats per minute.

The rate error was quantified as the mean absolute error between our sensor and reference respiration rates, MAE = $\frac{1}{N}\sum_{i=1}^{N}\left|f_{i}^{our}-f_{i}^{ref}\right|$, where *N* is the number of compared windows.

### 4.7 Joint Bending‐Angle Measurement and Calibration

For bending‐angle calibration, the dual‐gradient fiber was attached across the elbow or the knee together with two inertial measurement units (IMUs; BWT901CL, WitMotion) mounted on the limb segments on either side of the joint, and the LSL resistance and the IMU signals were recorded simultaneously during repeated flexion‐extension cycles. The bending angle was taken as the relative angle between the two IMUs. The relationship between bending angle and relative resistance change was fitted by least squares, using a quadratic function for the elbow and a linear function for the knee, with the goodness of fit quantified by the coefficient of determination (*R* ^2^).

To assess quantitative angle estimation, the fitted calibration was inverted to predict the bending angle from the measured resistance, and the predicted angle was compared with the IMU reference angle. For the elbow, whose quadratic calibration is monotonic and invertible above its turning point (θ ≈ 8°), the estimation accuracy was evaluated over the responsive flexion range (θ ≈ 10–74°); for the knee, whose linear calibration is monotonic over the entire range, the full measured range was used. The accuracy was quantified by the root‐mean‐square error, RMSE = [(1/N) Σ (θ_pred_ − θ_ref_)^2^]^0.5^, and the mean absolute error, MAE = (1/N)Σ|θ_pred_−θ_ref_|, where θ_pred_ and θ_ref_ are the estimated and reference angles and N is the number of samples.

### 4.8 Electrical Simulation

A numerical analysis method was developed for recognizing the percolation network and calculating the resistance in a nanowire network [39, 51, 52]. The method includes distributing CNT positions and angles, updating nanowire positions under uniaxial loading, and recognizing the percolation network and calculating resistance as shown in Figure S4(i‐iii). First, the CNT center position [*x_c_*, *y_c_*, *z_c_*] and orientation [θ,ϕ] are assigned based on azimuthal and polar angles of a spherical coordinate system. Perfect adhesion between CNT and the polymer matrix was assumed, and affine transformation was used to update the nanowire orientation and center positions when tensile stretching along the *x*‐axes was applied as follows

$$\left[x_{c},\;y_{c},\;z_{c}\right]=\left[\left(1+\varepsilon \right)x_{c0},\frac{y_{c0}}{\sqrt{1+\varepsilon }},\frac{z_{c0}}{\sqrt{1+\varepsilon }}\;\right]$$

$$\theta =\tan^{-1}\left[\frac{\tan\theta_{0}}{\left(1+\varepsilon \right)\sqrt{1+\varepsilon }}\right],\;\phi =\tan^{-1}\left[\tan\phi_{0}\frac{\sin\theta_{0}}{\sin\theta }\right]$$

where the subscript 0 represents the initial state, which is randomly distributed. The simulation allowed for periodic boundary conditions along the *y*‐ and *z*‐axes. Next, the minimum distance (*d*) between CNT pairs was computed to determine resistance (*R*) between CNTs. If the distance is less than the CNT diameter (*d_c_*), the resistance value has a contact resistance value; if the distance is greater than the CNT diameter and less than the tunneling cutoff distance, it has a tunneling resistance value. Lastly, the percolation network was identified using the depth‐first search algorithm, exploring linked clusters of CNT members. Kirchhoff's first law was utilized to determine voltage within the percolation network, and resistance calculation was based on the current flow within the network and the assigned voltage difference. To reduce the variation resulting from a random initial distribution, the simulation was repeated 10 times under identical conditions, and the average resistance value was used as the final value.

The scope of this numerical model is to provide a qualitative understanding of how filler concentration affects the piezoresistive behavior in the final fiber. Accordingly, the model assumes a random, isotropic filler distribution and does not explicitly simulate the complex physical phenomena of the TDP, such as process‐induced filler alignment. This assumption is supported by direct scanning electron microscopy (SEM) imaging of our fiber's cross‐section (Figure S10), which shows a largely random CNT network. We assume these results stem from the relatively slow drawing speed (0.5–1 m/min) and wide width, which resulted in low shear stress and consequently minimized the alignment effect. Carbon black was not modeled explicitly because the simulation was intended to capture the dominant strain‐sensitivity mechanism, namely the strain‐induced disruption of the high‐aspect‐ratio CNT percolation network that governs the resistance change. The low‐aspect‐ratio CB, which contributes mainly to baseline conductivity and local junctions and is present at the same CB:CNT ratio in both layers, affects the absolute response but not the sensitivity contrast between the layers; its role in conductivity and drawability was characterized experimentally.

### 4.9 Dataset

Our dataset consists of two distinct types: pretraining and fine‐tuning datasets, designed for different tasks, including gesture recognition, sign language interpretation, baseball movements, and piano playing. For each task, we collect sensor signals along with corresponding labels. Each class is recorded 20 times, consisting of 10 repetitive and 10 sequential instances to enhance generalization. The mean duration across all gesture classes was 1.0 ± 0.4 s. For the pretraining dataset, we split each class into training and validation sets with an 8:2 ratio. The best‐performing model on the validation set is selected for further fine‐tuning. During the transfer (fine‐tuning) stage, where a new user wears the glove, we collect a 5‐shot dataset (five repetitions per class action) to efficiently adapt the model to the new user's signals. To consider temporal correlations in signal patterns due to the low‐dimensional nature of the input, we group 64 consecutive signals using a sliding time window with a stride of 1. The signals are normalized using min‐max normalization. The zero class represents instances where no action is performed.

### 4.10 Model Structure and Training

To evaluate the effectiveness of our layered composite fiber in enriching information for the neural network, we designed an LSTM‐based [53] model for task identification (Figure S22). The architecture consists of a five‐layer LSTM with a hidden dimension of 64 and a dropout rate of 0.3. The windowed signal groups are encoded using LSTM layers to capture temporal correlations in the layered fiber signals. The final embedding vector is passed through a linear block, which includes a SiLU [54] activation function and a softmax output layer for classification. Additionally, a residual connection is established between the last signal input and the embedding vector before feeding it into the linear block. The model is trained using the cross‐entropy loss function, with the AdamW [55] optimizer and an initial learning rate of 1e‐4. For fine‐tuning, the pre‐trained model parameters are used to adapt to new users. With the 5‐shot transfer dataset, the model is fine‐tuned for 30 epochs using cross‐entropy loss and the AdamW optimizer with a learning rate of 3e‐4. Once fine‐tuned, the model is employed for real‐time inference of incoming signals.

### 4.11 Real‐Time Inference

Incoming real‐time signals are grouped into 64‐sample windows and passed through the fine‐tuned model for action classification. If the predicted action class is zero (no action), the system continues without intervention. If the predicted action class is nonzero, indicating an action has been detected, we apply a majority voting mechanism over five consecutive predictions to reduce misclassification at the start of an action. During this early prediction stage, the system can modify its output if necessary. After five action label predictions, the system displays the majority class output until a zero class is predicted, mitigating jittering in the output predictions. This approach ensures a stable and accurate classification of actions in real‐time scenarios.

### 4.12 Human Subjects and Ethics Statement

All experiments involving human participants were carried out in accordance with the relevant guidelines and regulations and were approved by the Institutional Review Board (IRB) of the Korea Advanced Institute of Science and Technology (KAIST) (approval no. KAISTIRB‐2026‐23). These experiments comprised wearable physiological monitoring (arterial pulse and respiration), joint and body‐motion measurements, and glove‐based gesture‐recognition data collection. Written informed consent was obtained from all participants prior to participation.

## Conflicts of Interest

The authors declare no conflicts of interest.

## Supporting information




**Supporting File 1**: adma74328‐sup‐0001‐SuppMat.docx.

**Supporting File 2**: adma74328‐sup‐0002‐MovieS1.mp4.

**Supporting File 3**:adma74328‐sup‐0003‐MovieS2.mp4.

**Supporting File 4**:adma74328‐sup‐0004‐MovieS3.mp4.

**Supporting File 5**:adma74328‐sup‐0005‐MovieS4.mp4.

**Supporting File 6**:adma74328‐sup‐0006‐MovieS5.mp4.

## Data Availability

The authors declare that the data supporting the findings of this study are available within the article and its Supplementary Information files. Extra data or source files are available from the corresponding author. The authors declare that protocols and codes are available through GITHUB: https://github.com/MinKim14/MultiGradientSensing
