# Supplementary material for: 1‐D Collocated Dual‐Gradient Sensory Fibers for Comprehensive Sensing and Decoding of Complex Human Motion and Physiology
Source: Adv Mater. 2026 Jul 27;38(53):e74328. doi: 10.1002/adma.74328 (PMC13591664; doi:10.1002/adma.74328)
Supplement: Supplementary file 1 [file ADMA-38-e74328-s003.docx]

**Supporting Information**

**1-D Collocated Dual-Gradient Sensory Fibers for Comprehensive Sensing and Decoding of Complex Human Motion and Physiology**

Yunheum Lee^1,†^, Sungha Jeon^1,2,†^, Min Kim^3,†^, Joonhee Won^4^, Jinwook Yeo^5^, Kyunghyun Jo^6^, Soyeon Lee^7^, Kieun Park^1^, Yechan Yang^8^, Chul Kim^1^, Gun-Hee Lee^9^, Seunghwa Ryu^5^, Sungho Jo^3^* and Seongjun Park^2,8,10,11,12,13,14^*

^1^ Department of Bio and Brain Engineering, Korea Advanced Institute of Science and Technology (KAIST), Daejeon, Republic of Korea

^2^ Medical Research Center, Seoul National University, Seoul, Republic of Korea

^3^ School of Computing, Korea Advanced Institute of Science and Technology (KAIST), Daejeon, Republic of Korea

^4^ Department of Materials Science and Engineering, Korea Advanced Institute of Science and Technology (KAIST), Daejeon, Republic of Korea

^5^ Department of Mechanical Engineering, Korea Advanced Institute of Science and Technology (KAIST), Daejeon, Republic of Korea

^6^ Program of Brain and Cognitive Engineering, Korea Advanced Institute of Science and Technology (KAIST), Daejeon, Republic of Korea

^7^ School of Electrical Engineering, Korea Advanced Institute of Science and Technology (KAIST), Daejeon, Republic of Korea

^8^ Interdisciplinary Program in Bioengineering, College of Engineering, Seoul National University, Seoul, Republic of Korea

^9^ Departments of Biomedical Engineering, Ulsan National Institute of Science and Technology (UNIST), Ulsan, Republic of Korea

^10^ School of Transdisciplinary Innovations, Seoul National University, Seoul, Republic of Korea

^11^ Department of Biomedical Science, College of Medicine, Seoul National University, Seoul, Republic of Korea

^12^ Department of Transdisciplinary Medicine, Seoul National University, Seoul, Republic of Korea

^13^ Interdisciplinary Program in Neuroscience and Genetic Engineering, College of Natural Sciences, Seoul National University, Seoul, Republic of Korea

^14^ AI Institute, Seoul National University, Seoul, Republic of Korea

†These authors contributed equally to this work.

*Corresponding authors: S.J. (shjo@cs.kaist.ac.kr), S. P. (seongjunpark@snu.ac.kr)

**This file includes:**

**Figure S1 to S31**

**Table S1**

**Movie S1 to S5**

**References**

**Figure S1. Thermal drawing setup and representative fiber geometry**

Photograph of the custom-built fiber drawing tower, cross-sectional images of the preform and thermally drawn fiber, and fiber diameter as a function of capstan speed.

**Figure S2. Mechanical flexibility and stretchability of thermally drawn fibers**

Photographs of fiber bundles and representative bending and stretching states, together with stress–strain curves of pristine SEBS fibers and SEBS/CB/CNT composite fibers.

**Figure S3. Complex viscosity of SEBS/CB/CNT composites with different filler concentrations**

Complex viscosity as a function of frequency for conductive polymer composites with different CB/CNT loadings.

**Figure S4. Temperature-dependent viscoelastic behavior of single-filler composites**

**a,** Temperature dependence of tan δ for SEBS/CNT composites containing 3, 4, and 5 wt% CNT

**b**, Temperature dependence of tan δ for SEBS/CB composites containing 20, 25, and 30 wt% CB

The dashed line indicates tan δ = 1, corresponding to the transition between viscous-dominant (tan δ > 1) and elastic-dominant (tan δ < 1) behavior. Increasing filler concentration decreases tan δ, indicating progressively greater elastic character and reduced melt fluidity. The results identify the processability limits of the individual filler systems and provide the basis for selecting the CB:CNT ratio used in the dual-gradient fiber design.

**Figure S5. Computational workflow for conductive-network simulation**

Schematic of the simulation procedure used to model strain-dependent electrical behavior, including nanofiller position update, percolation-state evaluation, and current calculation through the conductive network.

The calculation involved three steps: (1) recalculating the positions of the nanofillers as strain increases, (2) evaluating percolation status at interconnecting nodes between fillers (connection, tunneling, and disconnection), and (3) calculating total current through each node to determine resistance.

**Figure S6. Gauge factor as a function of CB/CNT concentration**

Gauge factor of strain sensors prepared with different CB/CNT concentrations.

**Figure S7. Post-overstrain resistance behavior of the high-sensitivity layer**

Resistance response of the high-sensitivity layer after deformation beyond its nominal operating range, showing recovery of electrical continuity upon strain release.

**Figure S8. Mechanical hysteresis of the dual-gradient fiber at large deformation**

**a,** Stress–strain hysteresis curves measured at 100%, 300%, and 500% deformation

**b,** Quantified hysteresis values of the fiber sensor at different deformation levels

**Figure S9. Frequency-dependent cyclic response and recovery of the HSL**

Relative resistance response (R/R0) of the high-sensitivity layer under cyclic strain of 5% amplitude applied at stepwise increasing frequencies from 0.5 to 9 Hz, with five cycles at each frequency (top axis). Up to approximately 5Hz, the HSL tracked both the loading and unloading phases with near-complete baseline recovery in each cycle; at higher frequencies, the response amplitude progressively attenuated and the baseline rose, reflecting the onset of viscoelastic lag and incomplete recovery within the shortened cycle period.

**Figure S10. Cross-sectional and surface morphology of the dual-gradient fiber**

SEM images of the fiber cross section and outer surface.

**Figure S11. Environmental stability of the dual-gradient fiber**

**a,** Resistance behavior of the conductive layers under relative humidity from 30% to 90%

**b,** Resistance behavior of the conductive layers after exposure to different liquids

**Figure S12. Effect of temperature on the dual-gradient sensor**

**a, Resistance of the HSL and LSL versus temperature (20 to 40°C), normalized to the value at 25°C (R/R₂₅°C).**

**b, Resistance response (R/R₀) versus applied strain for the HSL and LSL measured at 20, 30, and 40°C.**

Both layers exhibit a small positive temperature coefficient of resistance (TCR), with TCR_HSL = 0.278 %/°C and TCR_LSL = 0.157 %/°C, corresponding to baseline resistance changes of only a few percent across the entire 20 to 40°C range. The net positive sign reflects the competing temperature responses of the two fillers: carbon black contributes a positive TCR through thermal-expansion-induced changes in inter-particle contacts, whereas carbon nanotubes contribute a negative TCR through thermally activated hopping, so the carbon-black-dominant composition (CB:CNT = 4:1) yields a small net positive value. The larger TCR of the HSL is consistent with its sparser, near-percolation network being more responsive to perturbation, the same property that underlies its higher strain sensitivity. In (b), the strain-response curves nearly overlap across the three temperatures and the HSL retains a much higher sensitivity than the LSL at every temperature, so the HSL/LSL sensitivity hierarchy is preserved. Because the temperature-induced baseline change is small relative to the strain-induced response and the hierarchy is maintained, the dual-gradient fiber provides consistent strain-sensing performance over this temperature range.

**Figure S13.** **Sewability of the dual-gradient fiber**

Photographs showing textile integration of the fiber by sewing.

**Figure S14. Features of radial artery pulse**

**a,** Schematic showing features of the pulse waveform

**b,** Data for UT, RWTT, PPT, BPM, HT/HP, and HD/HP derived from 10 pulse waveforms

The percussion wave (P) signifies the initial pressure wave produced by the heart's contraction. The tidal wave (T) is the secondary upward movement, offering insights into arterial rigidity and peripheral resistance. The dicrotic wave (D) denotes the blood's rebound against the closed aortic valve and the subsequent forward blood movement in the arteries.

Systolic upstroke time (UT) measures the interval for the pressure wave to peak after heart contraction, providing information about blood ejection speed and arterial stiffness. Reflected wave transit time (RWTT) indicates the duration for the pressure wave to travel to peripheral areas and return to the heart, affected by arterial stiffness and the distance to the reflection site. Peak-to-peak time (PPT) measures the interval between the systolic peak (P) and the dicrotic peak (D), serves as a key indicator of arterial stiffness and pulse wave velocity.Total time (TT) represents the full duration of one heartbeat, providing information on heart rate and rhythm. The amplitude ratio of the reflected wave to the advancing wave (H_T_/H_P_) indicates arterial stiffness or peripheral resistance, while the amplitude ratio of the dicrotic wave to the advancing wave (H_D_/H_P_) offers insights into the arterial wall's elasticity and the behavior of the aortic valve.

**Figure S15. Minutes-scale stability and physiological fidelity of pulse monitoring**

**a, Photograph of the dual-gradient sensor woven into a wristband over the radial artery, together with the commercial PPG reference placed on the fingertip**

**b, Pulse signals recorded simultaneously over 90 s by the dual-gradient sensor (red, R/R₀, left axis) and the commercial PPG sensor (black, V/V₀, right axis)**

**c, Scatter plot of the beats-per-minute (BPM) values obtained from the peak-to-peak intervals of the two sensors, computed from the 103 peaks detected over the 90 s recording (R² = 0.975, MAE = 0.29 bpm)**

**d, Representative early (left) and late (right) waveform segments, showing the percussion (P), tidal (T), and diastolic (D) peaks.**

To evaluate whether the sensor can monitor the pulse stably over an extended period and whether its output correlates with the true pulse, we recorded the radial-artery pulse simultaneously with a commercial PPG reference for 90 s. A total of 103 peaks (mean heart rate 69.6 bpm) were reliably extracted from both sensors, and the per-interval BPM values from the two sensors were in close agreement (R² = 0.975, mean absolute error = 0.29 bpm). The characteristic percussion (P), tidal (T), and diastolic (D) features remained clearly resolved in both the early and late segments of the recording, indicating that the waveform retains physiologically meaningful information throughout the measurement and is suitable for cardiovascular-parameter extraction.

**Figure S16. Single-joint bending-angle calibration of the dual-gradient fiber**

**a, Simultaneous recording of the LSL response (blue, R/R₀) and the IMU-measured bending angle (black) during four repeated elbow flexion-extension cycles**

**b, Calibration of LSL R/R₀ against elbow bending angle, with a monotonic quadratic fit (R/R₀ = 0.000491θ² − 0.00793θ + 1.0365; R² = 0.994)**

**c, Same as (a) for the knee**

**d, Calibration for the knee, with a monotonic linear fit (R/R₀ = 0.0233θ + 1.032; R² = 0.967)**

To quantify the relationship between joint bending angle and sensor output, the dual-gradient fiber was attached together with a reference IMU across the elbow and the knee, and both signals were recorded during repeated flexion. The LSL response increased monotonically with the bending angle for both joints and was well described by a single-valued calibration (R² = 0.994 for the elbow and 0.967 for the knee), so the bending angle can be recovered from the resistance through this calibration. Evaluated against the IMU reference, the recovered angle had an RMSE of 2.5° and an MAE of 1.6° for the elbow over the responsive flexion range (θ ≈ 10–74°), and an RMSE of 3.6° and an MAE of 2.8° for the knee over the full measured range. The LSL is used for these large-amplitude joint motions because they exceed the high-sensitivity working range of the HSL.

**Figure S17.** **Wearable applications of dual-gradient fiber for biometric monitoring**

**a,** Resistance change of HSL during chest breathing movements.

**b,** Resistance change of LSL during knee squat movements.

**Figure S18. Minutes-scale stability and physiological fidelity of respiration monitoring**

**a, Photograph of the simultaneous measurement setup, in which the dual-gradient fiber woven into an abdominal band and a force-based commercial respiratory sensor were worn together**

**b, Respiration waveforms recorded simultaneously over 3 min by the dual-gradient sensor (red, R/R0) and the commercial sensor (black, V/V0)**

**c, Scatter plot of the breath-by-breath respiration rate measured by the dual-gradient sensor against the commercial sensor (36 peaks analyzed; R² = 0.996, MAE = 0.15 bpm)**

**d, Scatter plot of the per-breath peak relative amplitude measured by the two sensors (Pearson's r = 0.80)**

To validate the utility of the sensor for respiration monitoring, the dual-gradient fiber was worn together with a commercial respiratory sensor and the two signals were recorded simultaneously. The two signals exhibited synchronized peaks throughout the 3 min recording, and their breath-by-breath respiration rates were in close agreement (R² = 0.996, MAE = 0.15 bpm). The per-breath peak amplitudes were also strongly correlated (Pearson's r = 0.80), indicating that the dual-gradient sensor captures not only the timing but also the relative depth of each breath, and therefore carries sufficient physiological meaning for respiration monitoring.

**Figure S19. Schematic illustrations of commercial smart glove and fiber-based textile glove**

**a** Schematics for sensor placement and backend connection for a commercial smart glove

**b** Schematics for sensor placement and backend connection for fiber-type textile gloves.

In this case of fiber-based smart textile gloves, it is very difficult to distinguish signals using only a single sensor layer, but as the two sensors are merged, the signal distinguishing features increase and higher accuracy can be achieved.

**Figure S20. Voltage-divider circuit for dual-channel fiber readout**

Circuit configuration used for simultaneous measurement of the dual-gradient fiber.

**Figure S21. Single-joint contribution analysis of the single-fiber dual-gradient**

**a–c.** Representative HSL (red) and LSL (blue) relative resistance responses (R/R_0_) during controlled isolated bending of **a.** the proximal interphalangeal (PIP) joint, **b.** the metacarpophalangeal (MCP) joint, and **c.** the wrist, with the remaining joints held fixed. **d.** HSL-to-LSL peak-to-peak ratio (P_HSL_/P_LSL_) extracted from the single-joint responses, increasing from the PIP (1.01 ± 0.01) to the MCP (1.08 ± 0.01) to the wrist (1.18 ± 0.03) (mean ± s.d., n = 4 bending cycles). Because each joint imposes a different range of motion and local strain, bending it drives the fiber into a different strain regime; the HSL/LSL peak-to-peak ratio therefore acts as a position-dependent strain-regime fingerprint that distinguishes the mechanical contribution of each joint.

**Figure S22. Neural-network architecture for glove-based gesture classification**

Schematic of the neural network used to predict hand motion from smart-glove signals.

**Figure S23. Comparison of recognition accuracy across model architectures**

Classification accuracy obtained using different learning models.

**Figure S24. New-user fine-tuning performance for 21-class gesture recognition**

Inference accuracy across training epochs for a new participant.

**Figure S25. User interface for real-time sign-language translation**

Photograph of the custom-built ASL translation interface.

**Figure S26. Baseball pitch grips and corresponding hand configurations**

Representative photographs of baseball pitch grips and schematic illustrations of the associated hand motions.

**Figure S27. Confusion matrix for pitch-type classification**

Confusion matrix for classification of five baseball pitch types and the resting state.

**Figure S28. Piano-chord configurations used for glove-based classification**

Schematic illustration of representative piano-chord hand configurations.

**Figure S29. Confusion matrix for piano-chord classification**

Confusion matrix for classification of piano-chord hand postures.

**Figure S30. Schematic illustration of the preform fabrication process for multi-material preform.**

**a,** Fabrication method of the multi-layers, including a pure SEBS core rod, a pure SEBS film, and a conductive CB/CNT film

**b,** Fabrication steps involved in assembly of the preform

**Figure S31. Schematic illustration of the independent signal acquisition architecture**

The HSL and LSL are electrically isolated by an intervening SEBS insulation layer and are connected to separate electrode pairs. Consequently, each layer is read out through an independent channel (Channel 1: LSL, Channel 2: HSL), enabling simultaneous acquisition of two resistance signals without computational separation from a combined signal.

| **Ref.** | **Materials / structure** | **Fiber diameter / geometry** | **Fabrication method** | **Sensing channels per fiber** | **Gauge factor** | **Working strain range** | **Response / recovery time** | **Durability / robustness** | **Demonstrated applications** |
| --- | --- | --- | --- | --- | --- | --- | --- | --- | --- |
| Qu et al., 2022 | Crack-based CNT/TPU core–sheath fiber | Approximately  983μm | Coaxial wet spinning | 1 | GF = 4520 at 0–250%; 23186 at 250–400%; 66600 at 400–520% | Up to 520% | ~60 ms | 5000 tensile cycles at 80% strain | Pulse wave; swallowing; finger joint and wrist movement; wireless sign language translation |
| Wu et al., 2023 | APTES-MWCNTs/silicone rubber core–sheath fiber with double strain-layered crack structure | Core: 0.6 mm; sheath thickness: 0.3 mm | Coaxial wet spinning | 1 | GF = 2165 at 0–30%; 7381 at 25–30% | 0–30% | ~42 ms | >1000 cycles at 0–30% strain; hysteresis 7.08% | Respiratory rate; heart rate; cuffless blood-pressure estimation |
| Yue et al., 2024 | Porous CNTs/AgNPs/TPU composite fiber (CATF) | ~ 1.39 mm | Wet spinning | 1 | GF = 35.03 at 0–120%; 843.48 at 120–220%; 4295.05 at 220–248% | ~248% | 120/140 ms | 3000 cycles at 50% and 120% strain | Human motion monitoring; pronunciation recognition; gesture recognition; tactile sensing; photothermal conversion |
| Yao et al., 2025 | Closed-loop PAM/Ca-Alg hydrogel fiber with customized localized crack and Ecoflex encapsulation | Hydrogel fiber prepared using 0.9 mm capillary | Hydrogel fiber molding + closed-loop assembly + crack engineering | 1 | GF up to 3929.9 (NEEM); GF up to 1188.5 (PEEM) | 0.02–80% | 78/52 ms | 5000 cycles at 10% strain | Pulse-to-joint motion; wireless sign language recognition; 98.1% accuracy |
| Shao et al., 2025 | CNT@Ni-CNT core–sheath fiber | Core: 0.94 ± 0.04 mm; sheath: 0.11 ± 0.02 mm | Coaxial wet spinning | 1 | GF = 81.58 | Up to 190% strain sensing; fracture strain 210% | 172/258 ms at 10% strain | >30,000 cycles at 10% strain, 1 Hz | Human motion monitoring; EMI shielding |
| Wu et al., 2025 | AgNP-coated PDA-modified double-covered yarn | ~0.75 mm | PDA modification + AgNP reduction | 1 per yarn; multiple distributed yarns in clothing | GF = 92.2 at 0–70%; 464.3 at 70–130%; 906.4 at 130–170%; average GF = 414 | Up to 170% | 130/190 ms | 1000 cycles at 50%; fabric 600 cycles at 30%; washing tested | COPD respiratory monitoring; Parkinson’s disease monitoring; 96.67% accuracy |
| Zhang et al., 2026 | CNT/graphene hybrid core–shell Euler spiral fiber | Diameter: N.R.; shell-to-core diameter ratio = 1.15 for optimized fiber; Euler spiral pitch P = 0.5–1 mm | Coaxial wet spinning + mechano-patterned spiral coating | 1 | GF ≈ 9.0 | 0–200%; up to 300% limit strain | ~200/190 ms | 10,000 cycles at 20% strain | Wearable electronics; soft sensing; programmable two-stage failure / electrical warning |
| **This work** | **CB/CNT/SEBS monolithic coaxial dual-gradient fiber** | **Tunable thermally drawn fiber diameter: sub-mm to ~1 mm** | **Monolithic preform-to-fiber co-drawing** | **2 independent collocated HSL/LSL channels** | **HSL: 51.28 at 0–10%, 752.31 at 20–30%; LSL: 12.42 at 0–10%, 34.99 at 80–100%** | **HSL ~40%; LSL ~160%** | **~150 ms** | **10,000 cycles at ~10%** | **Pulse; respiration; joint motion; superimposed deformation; single-fiber glove gesture decoding; ASL; baseball pitch; piano chord recognition** |

**Table S1. Comparison of representative fiber-based strain sensors**

Comparison of representative fiber-based strain sensors for wearable motion and physiological monitoring. Values are listed as reported in each reference; N.R., not reported.

**Movie S1. Fabrication of dual-gradient sensory fibers via a high-precision thermal drawing process (TDP).**

This movie highlights the scalable manufacturing of the collocated dual-gradient sensory fiber using a custom-built TDP tower. The process demonstrates the precise reduction of a macroscopic multi-material preform into hundreds of meters of functional 1-D fiber while maintaining the complex internal coaxial architecture.

**Movie S2. Real-time American Sign Language translation of the word "BMIL" using a single-fiber interface.**

This movie demonstrates the high-precision translation of American Sign Language into text, focusing on the sequence of letters forming the word "BMIL".

**Movie S3. Real-time American Sign Language translation of the sentence “LIFE IS A GIFT" using a single-fiber interface.**

This movie demonstrates the robust decoding capabilities of our hardware-to-AI platform through the continuous translation of a complex sentence: "LIFE IS A GIFT".

**Movie S4. Classification of baseball pitch types based on finger grip configurations using a single fiber.**

This movie demonstrates the platform's ability to discriminate between distinct baseball pitch types—such as fastballs, sliders, and curveballs—by capturing the subtle variations in finger grip and hand kinematics.

**Movie S5. Virtual music interface for chord-based piano performance via hand posture recognition.**

This movie demonstrates an advanced human–machine interface (HMI) that translates specific hand postures into musical chords in real-time. By utilizing the single-fiber sensory platform, the system enables a virtual piano performance.

**References**

1	Qu, X. *et al.* Crack-based core-sheath fiber strain sensors with an ultralow detection limit and an ultrawide working range. *ACS Appl. Mater. Interfaces* **14**, 29167-29175 (2022). https://doi.org/10.1021/acsami.2c04559

2	Wu, S. *et al.* An ultra-sensitive core-sheath fiber strain sensor based on double strain layered structure with cracks and modified MWCNTs/silicone rubber for wearable medical electronics. *Compos. Sci. Technol.* **231**, 109816 (2023). https://doi.org/10.1016/j.compscitech.2022.109816

3	Yue, X. *et al.* Tunable porous fiber-shaped strain sensor with synergistic conductive network for human motion recognition and tactile sensing. *Chem. Eng. J* **491**, 151853 (2024). https://doi.org/10.1016/j.cej.2024.151853

4	Yao, D. *et al.* Ultrasensitive and breathable hydrogel fiber‐based strain sensors enabled by customized crack design for wireless sign language recognition. *Adv. Funct. Mater.* **35**, 2416482 (2025). https://doi.org/10.1002/adfm.202416482

5	Shao, Y. *et al.* Core-sheath structured CNT@ Ni-CNT fiber-based multifunctional fabric with high-sensitivity, wide-range strain sensing, and enhanced electromagnetic shielding absorption. *Chem. Eng. J* **512**, 162358 (2025). https://doi.org/10.1016/j.cej.2025.162358

6	Wu, L. *et al.* Interlocking integration of elastic strain sensor yarn enables smart and distributed sensing in healthcare clothing. *Sci. China Mater.* **68**, 2079-2088 (2025). https://doi.org/10.1007/s40843-025-3372-6

7	Zhang, J. *et al.* Interfacial engineering of biomimetic Euler spiral fiber toward high-performance flexible strain sensors. *Appl. Surf. Sci.*, 166250 (2026). https://doi.org/10.1016/j.apsusc.2026.166250
